# Deep Phospho- and Phosphotyrosine Proteomics Identified Active Kinases and Phosphorylation Networks in Colorectal Cancer Cell Lines Resistant to Cetuximab

**DOI:** 10.1038/s41598-017-10478-9

**Published:** 2017-09-05

**Authors:** Yuichi Abe, Maiko Nagano, Takahisa Kuga, Asa Tada, Junko Isoyama, Jun Adachi, Takeshi Tomonaga

**Affiliations:** 1 Laboratory of Proteome Research, National Institute of Biomedical Innovation, Health and Nutrition, Ibaraki, Osaka, 567-0085 Japan; 2 Department of Biochemistry and Molecular Biology, Kyoto Pharmaceutical University, Yamashina-ku, Kyoto, 607-8414 Japan

## Abstract

Abnormality in cellular phosphorylation is closely related to oncogenesis. Thus, kinase inhibitors, especially tyrosine kinase inhibitors (TKIs), have been developed as anti-cancer drugs. Genomic analyses have been used in research on TKI sensitivity, but some types of TKI resistance have been unclassifiable by genomic data. Therefore, global proteomic analysis, especially phosphotyrosine (pY) proteomic analysis, could contribute to predict TKI sensitivity and overcome TKI-resistant cancer. In this study, we conducted deep phosphoproteomic analysis to select active kinase candidates in colorectal cancer intrinsically resistant to Cetuximab. The deep phosphoproteomic data were obtained by performing immobilized metal-ion affinity chromatography-based phosphoproteomic and highly sensitive pY proteomic analyses. Comparison between sensitive (LIM1215 and DLD1) and resistant cell lines (HCT116 and HT29) revealed active kinase candidates in the latter, most of which were identified by pY proteomic analysis. Remarkably, genomic mutations were not assigned in most of these kinases. Phosphorylation-based signaling network analysis of the active kinase candidates indicated that SRC-PRKCD cascade was constitutively activated in HCT116 cells. Treatment with an SRC inhibitor significantly inhibited proliferation of HCT116 cells. In summary, our results based on deep phosphoproteomic data led us to propose novel therapeutic targets against cetuximab resistance and showed the potential for anti-cancer therapy.

## Introduction

Protein kinases are key regulators of the phosphorylation signaling pathway, such as EGFR signaling pathway that controls various types of cellular functions, including cell cycle and cell movement^1^. Therefore, dysregulation of kinases is closely related to the hallmarks of cancer^2^. 518 kinases that are encoded in the human genome are defined as the “kinome”^1^. Analyses of the kinome provide essential insights into the relationship with cancer development. Previous genomic analyses revealed several point mutations on some kinase genes as cancer driver and mechanistic insights for intrinsic and acquired resistance to anti-cancer drugs^3, 4^. Moreover, gene fusion caused by genomic instability can form chimeric kinases, such as EML4-ALK^5^. Such chimeric kinases reorganize the cellular phosphorylation status, leading to development of characteristic subtypes in cancer^6^. These facts suggest that global analysis of the kinome by using omics approaches should provide information about anti-cancer druggable targets and their sensitivity to those drugs, which should contribute to overcoming drug-resistant cancers.

Although genomic analysis has provided several significant findings such as the identification of driver genes including many kinases in cancer, mechanisms for anti-cancer drug resistance cannot be fully explained by using genomic approaches. For example, modulation of phosphorylation signals by bypass pathways or aberrant localization of kinases, such as nuclear localization of EGFR, have been reported as reasons for drug resistance^7, 8^. Thus, proteomics approaches, as well as genomic approaches, are important for characterizing the kinome status.

Proteomic methods, particularly phosphoproteomics using immobilized metal affinity chromatography (IMAC)^9^, metal oxide affinity chromatography^10^, and hydroxyl acid-modified metal oxide chromatography^11^ have been widely applied to analyze the global phosphorylation status regulated by the kinome. In protein phosphorylation of serine, threonine, and tyrosine residues, phosphotyrosine (pY) residues in particular have been reported to have an important role in tumorigenesis^12^. Therefore, there have been many efforts to develop anti-cancer drugs targeting pY signaling. However, the depth of pY proteomics is limited because the percentage of pY peptides in all identified phosphopeptides is quite small (~2%) due to the low abundance of pY sites relative to phosphoserine and phosphothreonine sites^13^. To overcome the difficulty in analysis of pY signaling, we developed a highly sensitive pY proteomic analytical method and revealed an unknown pY signaling network^14^. Moreover, the combination of IMAC-based phosphoproteomics and deep pY proteomics may contribute in elucidating novel druggable targets that cannot be identified using genomic approaches.

**Figure 1:**
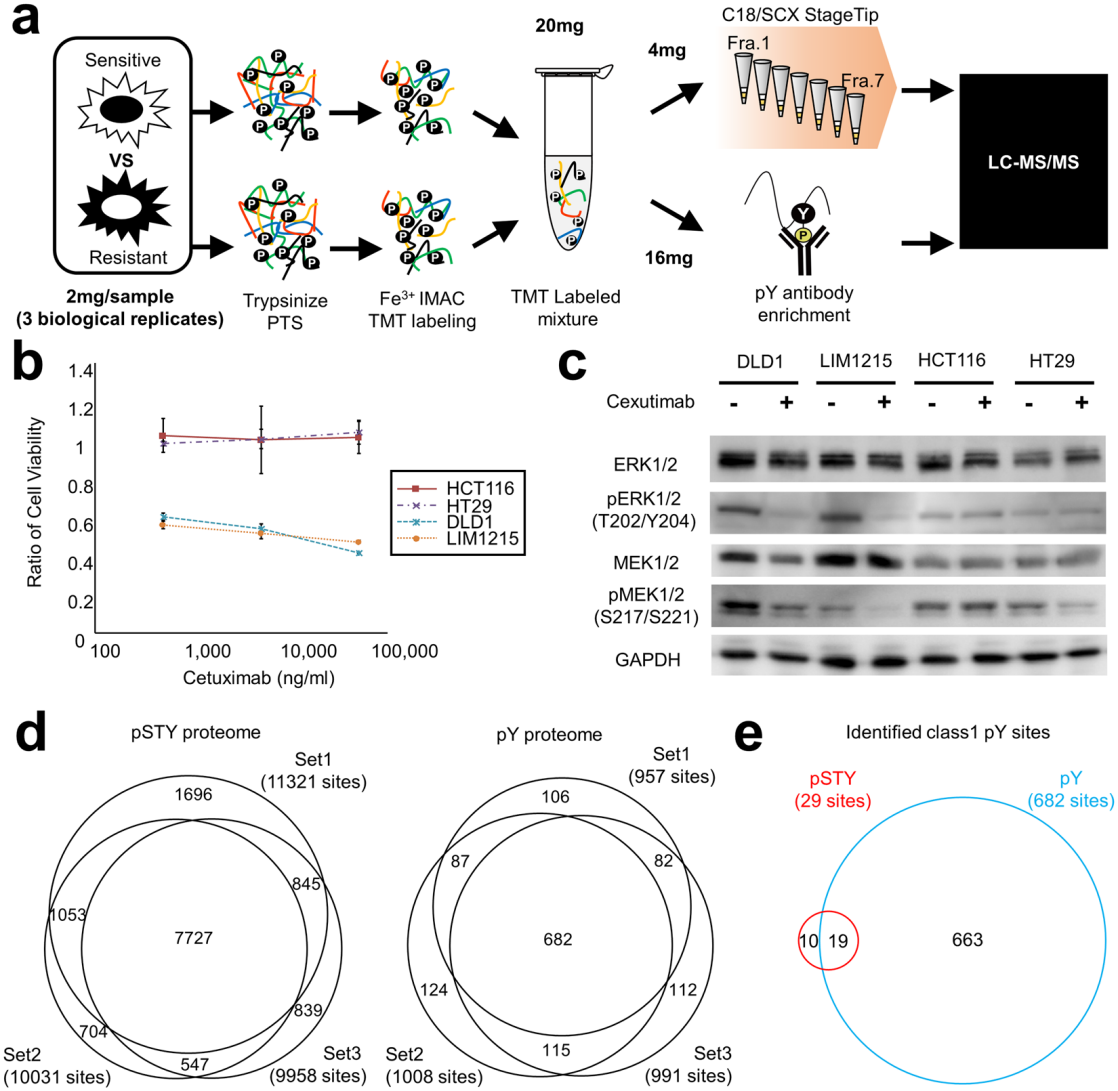
Phosphoproteomics of colorectal cancer cell lines that are sensitive or resistant to cetuximab. (**a**) Experimental flowchart in this study. (**b**) Cell viabilities of cetuximab-treated cell lines were obtained by cell growth assay. Error bars show SDs; N = 3. (**c**) Comparison of activation statuses of kinases in the EGFR signaling pathway between colorectal cell lines with or without cetuximab treatment. Total and phosphorylated ERK1/2 and MEK 1/2 levels were analyzed by western blotting. GAPDH was used as the internal control. (**d**) Identification of phosphorylation sites by phospho- and phosphotyrosine (pY) proteomics. Venn diagrams show class 1 phosphosites identified from IMAC-based phosphoproteomics analysis (Red) and pY sites identified from pY proteomics analysis (Blue). Each proteomic analysis was performed in triplicate. (**e**) Identification of phosphotyrosine sites by phospho- and phosphotyrosine proteomics. Venn diagrams show pY sites identified from pSTY phosphoproteomics (Red) and identified from pY proteomics (Blue). Plotted pY sites in the Venn diagram were identified in all experiments of each triplicate.

In this study, we performed deep phosphoproteomic analysis using cetuximab-sensitive and -resistant colorectal cancer cell lines and searched for active kinase candidates in the resistant cell lines as novel drug targets. To obtain deep phosphoproteomic information, we combined global phosphoproteomics (depicted as pSTY proteomics in Fig. 1a) with Fe^3+^ IMAC and pY proteomics (depicted as pY proteomics in Fig. 1a), and immunoaffinity enrichment of pY peptides. Then, from the deep phosphoproteomic data, we attempted to identify active kinase candidates and reconstruct an activated phosphorylation network by using Kinase–Substrate Relationships (KSRs) in resistant cell lines. Finally, we verified the effect of siRNAs or specific inhibitors of the candidates on cell growth of resistant cell lines and demonstrated the superiority of our strategy, which is based on deep phosphoproteomic data combined with a large amount of information on the pY status, for discovery of activated kinases in treatment-resistant cancer.

## Results

### Phosphoproteomic analysis of colorectal cancer cell lines that are sensitive or resistant to cetuximab

Deep phosphoproteomic data are essential for profiling activity of cellular kinome to overcome resistance to receptor TKIs. Therefore, we combined deep pY proteomic and IMAC-based global phosphoproteomic analyses to obtain a large amount of phosphoproteomic data in colorectal cancer cell lines.

Figure 1a shows the workflow of quantitative proteomic analysis that compared two cetuximab-sensitive cell lines and two cetuximab-resistant cell lines. We cultured and assayed colorectal cancer cell lines in Dulbecco’s modified Eagle’s medium (DMEM) supplemented with 1% fetal bovine serum (FBS) because many growth factors that are present in 5% FBS medium hamper the effect of cetuximab on sensitive cell lines (Figure S1). Although treatment with cetuximab clearly inhibited proliferation of DLD1 and LIM1215 cells, it did not affect growth of HCT116 and HT29 cells, even when a high concentration (50 μg/ml) of cetuximab was added (Fig. 1b). Therefore, we defined DLD1 and LIM1215 cells as the cetuximab-sensitive group and HCT116 and HT29 cells as the cetuximab-resistant group.

Because cetuximab is an anti-EGFR antibody, we first analyzed the activity status of kinases in the EGFR signaling pathway in each group. We found that treatment with 5 μg/ml cetuximab inhibited phosphorylation of downstream kinases (T202/Y204 on ERK1/2 and S217/S221 on MEK1/2) in the cetuximab-sensitive group, but exerted inconsistent responses in the cetuximab-resistant group (e.g., treatment with cetuximab decreased the expression level of pMEK1/2 in HT29 but not in HCT116; Fig. 1c). These results demonstrated that phosphorylation signaling after treatment with cetuximab was different between the cetuximab-resistant cells.

To elucidate activated kinases in each resistant cell line, we performed deep phosphoproteomic analysis using the cetuximab-sensitive and -resistant colorectal cancer cell lines. We used 4.0 and 16.0 mg of protein lysate for pSTY and pY phosphoproteomic analysis, respectively, as shown in Fig. 1a. In triplicate experiments, the pSTY proteomic analysis identified a total of 13,411 class 1 phosphosites and the pY proteomic analysis identified 1,308 class 1 pY sites (Fig. 1d). We found that 5.2% of the phosphosites (699 pSTY sites out of 13,411 sites) in the pSTY proteomics and 13.1% of the phosphosites (172 pY sites out of 1,308 sites) in the pY proteomics were unassigned in the PhosphositePlus database^15^ (Figure S2, Tables S1, S2), which indicated that our combinatorial approach could obtain information that may reveal unknown phosphorylation signaling. For further statistical analysis, we used 7,727 pSTY sites and 682 pY sites that were designated as class 1 sites in all experiments of each triplicate. As expected, the percentage of identified pY sites from the pSTY proteomics was low (<1%, 29 pY sites out of 7,727 pSTY sites) and most of the 682 pY sites detected in this study were only identified by the pY proteomic approach (Fig. 1e). These results indicate that the combination of pSTY and pY phosphoproteomic analyses is an efficient approach for the identification of deeper phosphorylation status of serine, threonine, and tyrosine residues.

### Identification of activated kinases in cetuximab-resistant cells from the results of deep phosphoproteomic data

**Figure 2:**
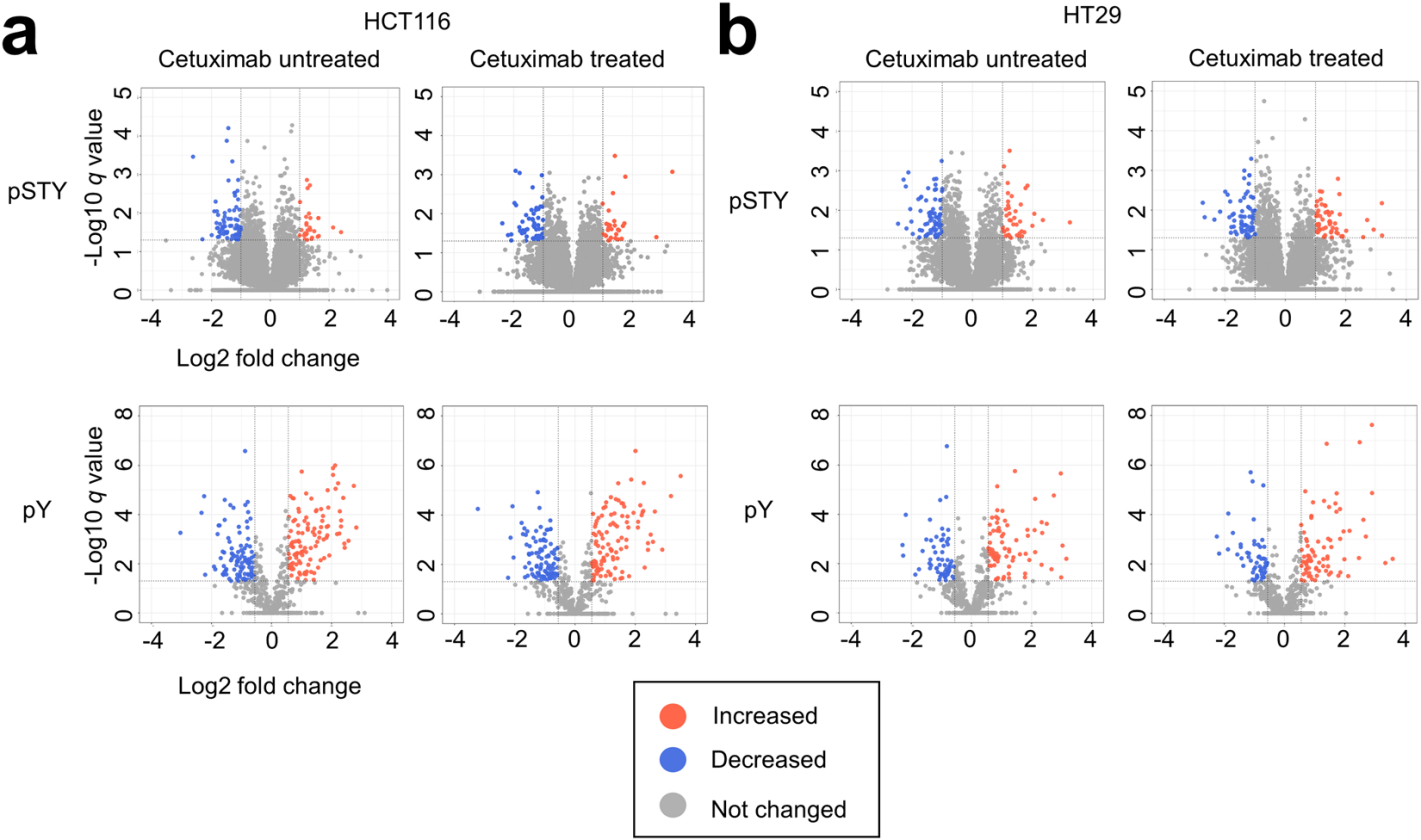
Volcano plot of phosphoproteomic data. (**a**,**b**) Volcano plots are depicted with the fold change of each phosphosite and the *q* value was calculated by performing a Welch’s *t*-test and a permutation test. The averages of the phosphoproteomic expression data of each resistant group cell line (**a**: HCT116, N = 3, b: HT29, N = 3) were compared with the averages of the data for each sensitive group cell line (DLD1 and LIM1215, N = 6). Red circles show phosphosites which have significant increases. Blue circles show phosphosites which have significant decreases. Gray circles are phosphosites without any differences. Results for HCT116 (**a**) and HT29 cell lines (**b**) with or without treatment of cetuximab by using pSTY proteomic data and pY proteomic data are shown.

Activity profiling of the kinome is important for an efficient anti-cancer treatment based on prediction of drug sensitivity. Therefore, to identify activated kinases in cetuximab-resistant cells from deep phosphoproteomic data, we first searched for phosphosites that showed significant differences between the cetuximab-sensitive and -resistant groups. We defined cutoff values in terms of fold change and statistical significance for discrimination between significant and insignificant differences in the quantitative results. The cutoff criterion of fold change was defined based on an experimental error of two standard deviations (SDs) calculated by using the reference samples. The average two-SD values for the triplicate pSTY and pY proteomic analyses were 0.989 and 0.556, respectively. Based on these data, we set the cutoff of fold change in the pSTY proteomics to 1.985 and that in the pY proteomics to 1.470. The cutoff of statistical significance was the *q* value (*q* < 0.05). Based on these two thresholds, we searched for phosphosites with significant increases in HCT116 and HT29 cells relative to the average of values in the cetuximab-sensitive cell group (Fig. 2a and b). The phosphosites are summarized in Tables S3 (pSTY proteomics) and S4 (pY proteomics).

**Figure 3:**
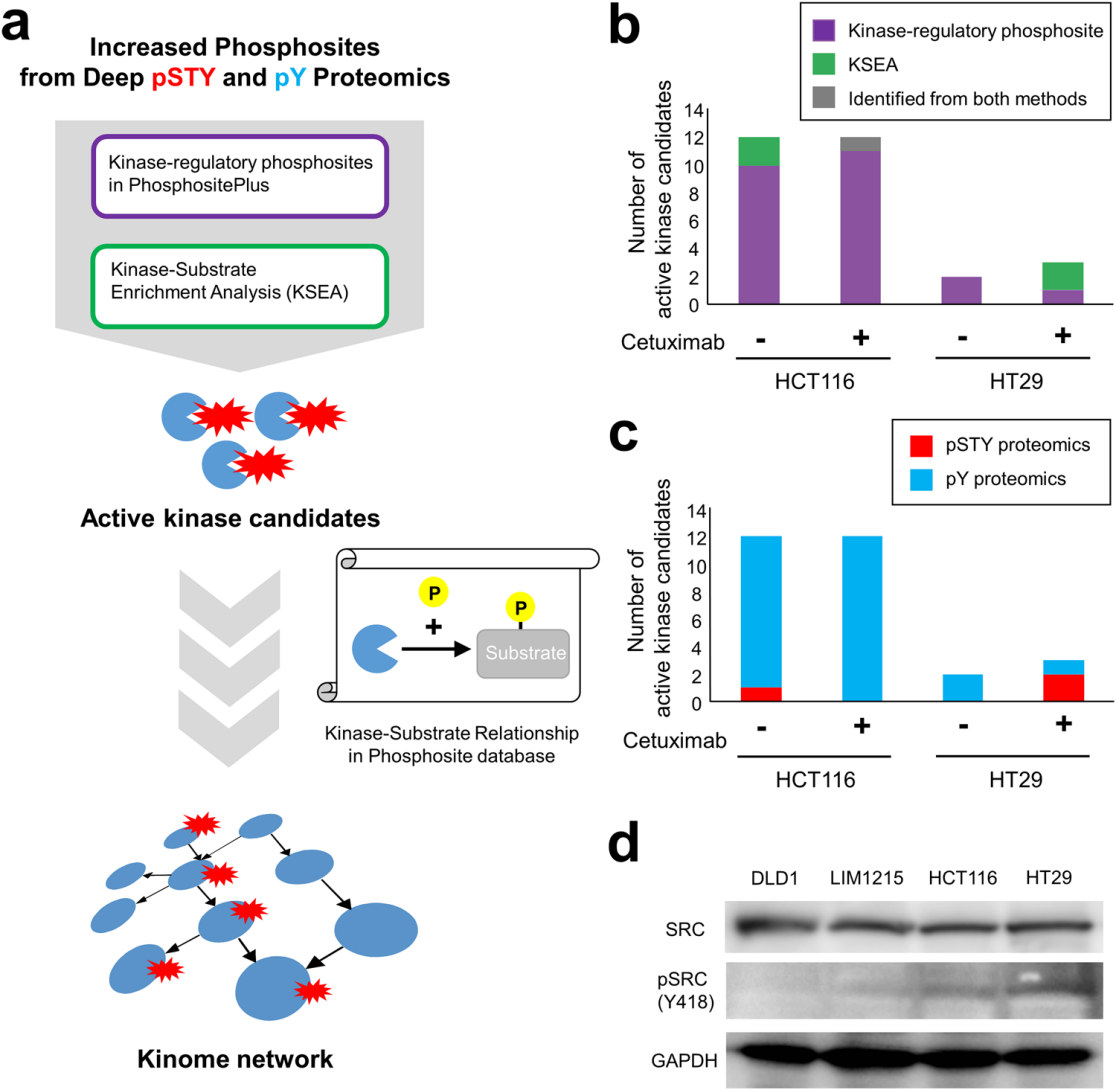
Identification of active kinase candidates as potential drug targets from the phosphoproteomic data. (**a**) Procedure for reconstruction of the kinase network. Active kinase candidates were predicted from increased phosphosites in deep pSTY or pY proteomic data by using the functional information for each phosphosite registered in the PhosphositePlus database or by KSEA prediction. By combining the active kinase candidates based on empirically validated KSRs in the PhosphositePlus database, the kinome networks were constructed. (**b**) Numbers of active kinase candidates obtained from two different approaches. Bar Graphs show the number of active kinase candidates in HCT116 cells and HT29 cells treated or untreated with Cetuximab. Purple: fraction of activated kinases identified by their phosphorylation statuses. Green: fraction of activated kinases predicted by KSEA. Gray: fraction of activated kinases identified by both methods. (**c**) Number of activated kinase candidates obtained from different proteomic data. Red: fraction of activated kinases identified from pSTY proteomic data. Cyan: fraction of activated kinases identified from pY proteomic data. (**d**) Verification of kinase activity by western blotting. The activation status of SRC in HCT116 and HT29 cells was analyzed by western blotting. GAPDH was used as an internal control.

From the results of the phosphosites with significant differences, we attempted to identify candidates of active kinases in each resistant cell line (HCT116 or HT29) and to reconstruct a network of those kinases. An outline of the procedures from identification of potential activated kinases to reconstruction of the kinase network is shown in Fig. 3a.

We employed two approaches to search for activated kinases. First, we focused on phosphorylation sites modified on kinases because it is widely known that activity of many kinases is regulated by the phosphorylation status itself (autophosphorylation)^12^. In HCT116 cells, 31 and 29 phosphorylation sites on kinases were significantly increased when treated or not treated with Cetuximab, respectively (Table S5). From the results for HT29 cells, 15 and 19 phosphorylation sites on kinases were also increased when treated or not treated with Cetuximab (Table S5). Next, we manually checked the function of the upregulated phosphorylation sites and identified kinase-regulatory phosphorylation sites that regulate the enzymatic activity of the kinases to select activated kinases in the resistant cell lines (Fig. 3b). Information about functions of each phosphorylation site was obtained from the Uniprot database^16^.

Second, we predicted active kinases from the whole phosphoproteomic data, including the pSTY and pY proteomics, by using a bioinformatic method. We applied kinase–substrate enrichment analysis (KSEA) for prediction of kinase activity to identify activated kinases in resistant cell lines compared to the activities in sensitive cell lines^17^. When untreated, we predicted the activation of two kinases in HCT116 cells. In addition, after 24 hours Cetuximab treatment, we predicted activation of one kinase (Fig. 3b). We verified the phosphorylation status related to enzymatic activation of those kinases by performing western blotting and revealed that one of the increased phosphorylation sites (Y418) on SRC was evaluated (Fig. 3d) even though protein expression of SRC was equal between the sensitive and resistant cell lines.

In total, 15 and 4 candidates of active kinases were identified from HCT116 cells and HT29 cells (Table 1). Noteworthy, most of the phosphosites on kinases (>70% in all conditions) were identified only from pY proteomics data (Fig. 3c), indicating that analysis with pY proteomics could contribute for activity profiling by means of the phosphorylation status of kinases.

**Table 1:** Summary of active kinase candidates that were regulated by autophosphorylation (“Kinase-Regulatory Phosphosite on Kinase” in the table), potential activated kinases predicted by the KSEA algorithm (“KSEA” in the table), upstream kinases connected with those activated kinases via KSRs (“Upstream Kinase of Kinase-Regulatory Phosphosite” in the table), and point mutations of identified kinases annotated in the COSMIC database (“Mutation” in the table).

Cell line	Kinase	Kinase-regularoty Phosphosite on Kinase	KSEA	Upstream Kinase of Kinase-regularoty Phosphosite	Mutaion
HCT116	ABL1	Y393		HCK, LYN	Y257C
	CDK12	T893		CDK7	
	HCK	Y411			
	JAK2		○		
	LCK	Y394			R484W
	LYN	Y397	○		
	MAP2K6		○		
	MAPK1	Y187		ABL1, JAK2, MAP2K1, RET	
	MAPK12	Y185			
	MAPK13	Y182			
	MAPK14	Y182		MAP2K3, MAP2K4, MAP2K6, MAP3K5, MAP3K6, RET	
	MAPK3	Y204		JAK2, MAP2K1, MAP2K2, RET	
	PRKCD	Y313, Y334			
	SRC	Y419c		PDGFRA, PDGFRB, PKACA	
	YES1	Y426		SRC	
HT29	CDK1		○		
	DYRK4	Y264			
	MAPK1	Y187		ABL1, JAK2, MAP2K1, RET	
	MAPK3		○		

In addition, we analyzed the presence of genomic mutations in the candidates of active kinases in the cell lines used in this study by using COSMIC database^18^. However, mutations were not identified in most of those kinase genes except for ABL1 and LCK. This finding indicates that the phosphoproteomic approach in this study contributed to identification of novel candidates of activated kinases that could not be analyzed by using genomic approaches.

### Construction of a kinase network by using KSRs

**Figure 4:**
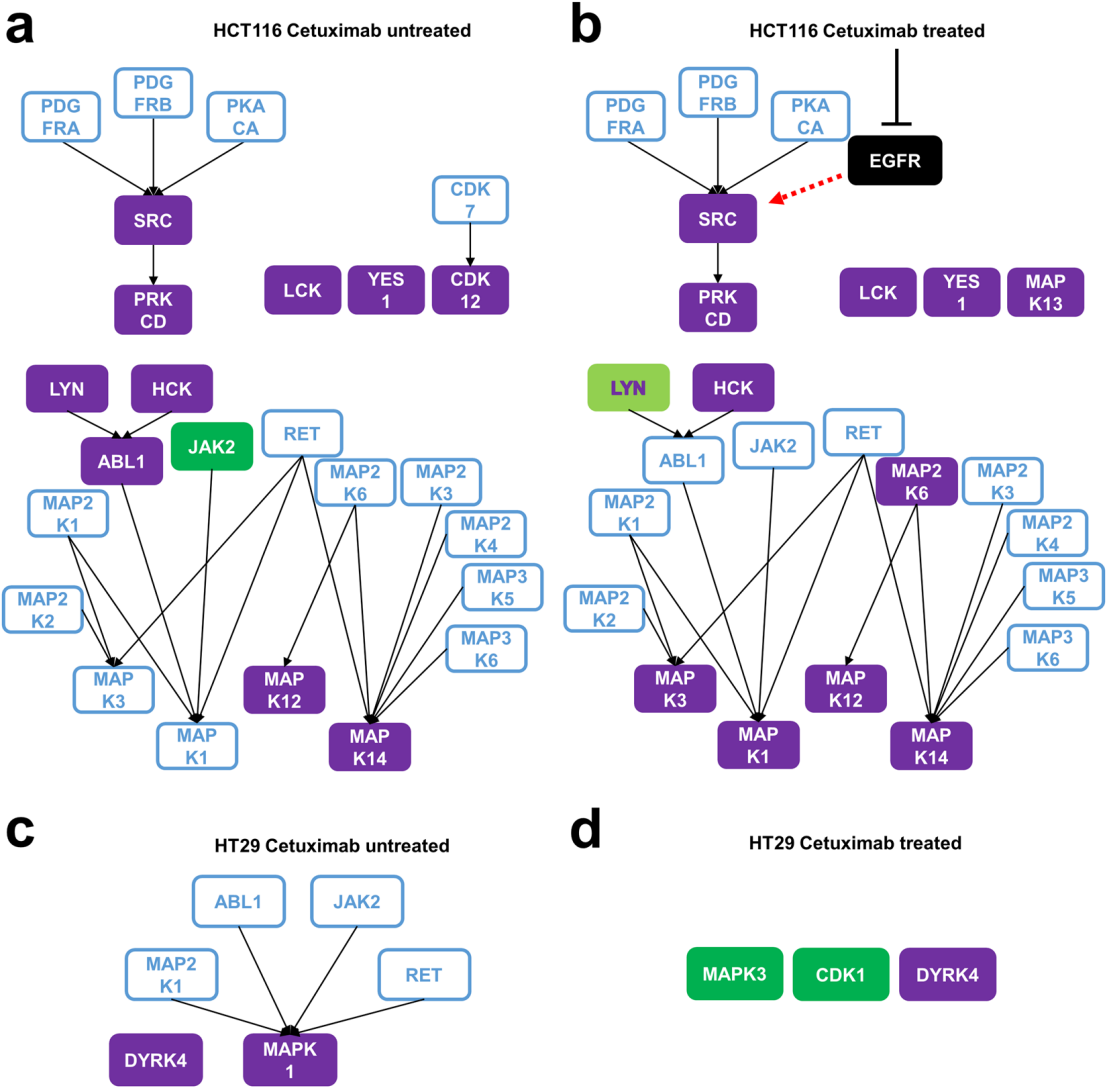
Activated phosphorylation network reconstructed from phosphoproteomic data. (**a**–**d**) Activated phosphorylation network in HCT116 (**a**,**b**) and HT29 cells (cd, **d**) were constructed by connecting the activated kinases shown in Fig. 3 with KSR assigned in the PhosphositePlus database. Purple rectangles: activated kinases identified by their phosphorylation statuses. Green rectangles: activated kinases predicted by KSEA. Purple character and light green rectangle (Lyn at 24 hours): kinases identified by the combination of phosphorylation status and KSEA algorithm. Blue rectangles with a white background: upstream kinases of activated kinases connected by KSR in PhosphositePlus.

It has been reported that rewiring of the phosphorylation signaling is one of the reasons for drug resistance in anti-cancer therapy^4^. For example, constitutive activation of kinases in downstream of therapeutic targets or bypass signaling caused by unexpected activation of other kinases is responsible for (or involved in) drug resistance^4^. To discuss mechanistic insights about a rewired signaling cascade in colorectal cell lines intrinsically resistant to Cetuximab, we constructed a network of phosphorylation signaling. We used the active kinase candidates as network nodes and connected them with experimentally validated KSRs that were registered in the PhosphositePlus database^15^. Figure 4a and b show the phosphorylation network in HCT116 cells by connecting 13 activated kinases with their 21 KSRs. In the networks, phosphorylation signaling from SRC to PRKCD was activated with and without cetuximab treatment. In HT29 cells, activated phosphorylation networks were quite limited because of an insufficient number of activated kinases (Fig. 4c,d).

### Inhibition of activated kinases affect cell proliferation in resistant cells

**Figure 5:**
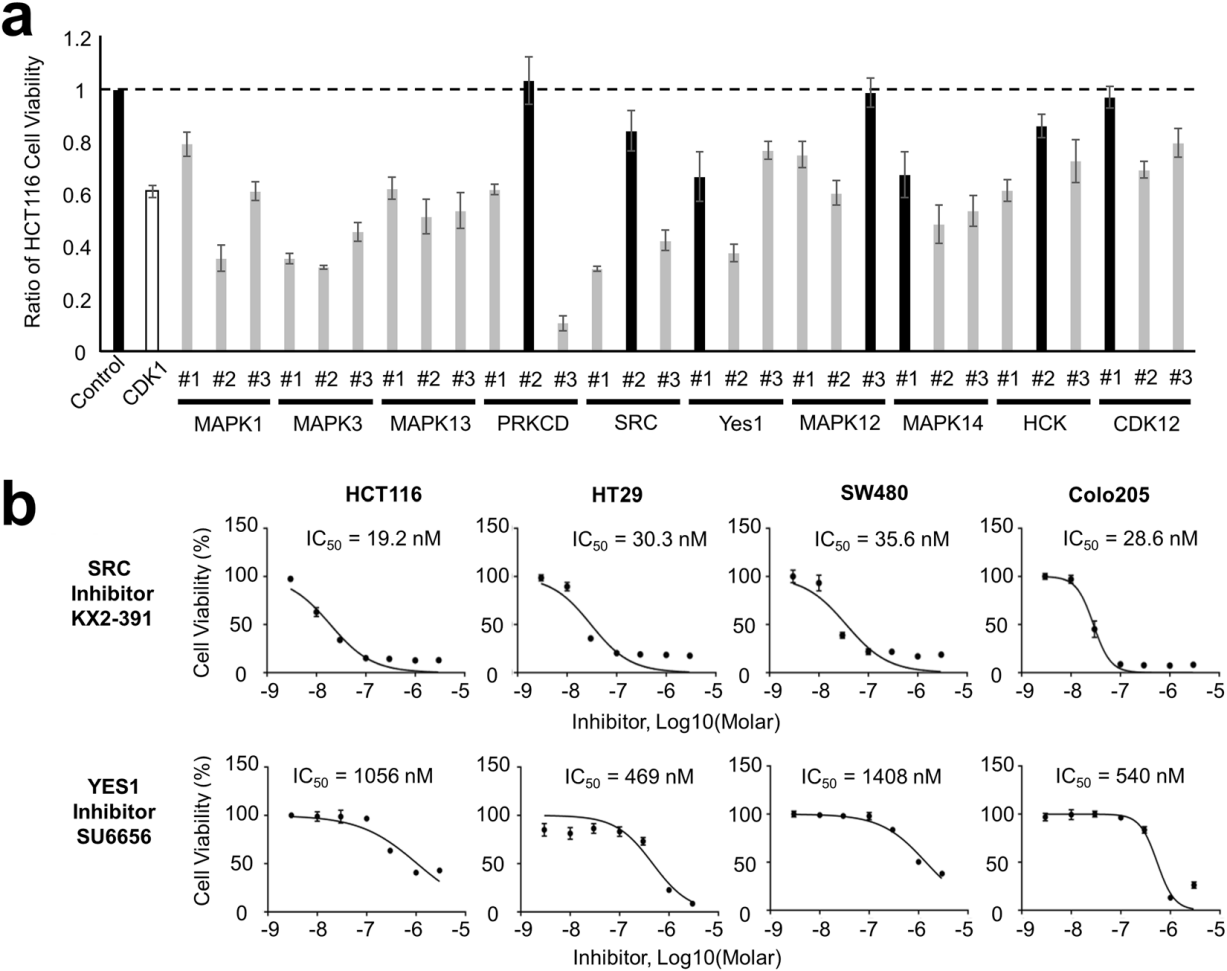
The effect of the knockdown of activated kinase candidates on cell proliferation. (**a**) Viability of HCT116 cells was analyzed following treatment with three siRNAs (#1, #2, and #3) specific to each activated kinase summarized in Table S7. Gray bars show results of siRNAs that had a significant effect (*q* < 0.05) on cell proliferation. Black bars show results of the control siRNA and siRNAs without a significant effect (*q* > 0.05). White bars show the results of CDK1 siRNA that was a positive control of the siRNA experiment. Error bars show SDs; N = 3. (**b**) Cell viability of cetuximab-resistant cells under treatment with two TKIs targeting SRC (KX2-391) and YES1 (SU6656) in a dose-dependent manner. Curve fitting was performed to the data from the growth assay. Error bars show SDs; N = 3.

Finally, we validated the functional roles of the active kinase candidates in the cell growth of HCT116 cells. We treated HCT116 cells with three siRNAs for each kinase and confirmed the efficiency of the siRNAs by quantitative reverse-transcription polymerase chain reaction (qRT-PCR) (Table S6). We observed that the knockdown of three kinases (MAPK1, MAPK3, MAPK13) led to a significant decrease in cell proliferation with all of three siRNAs to each kinase (Fig. 5a, Table S7). Additionally, two out of three siRNAs targeting six kinases (CDK12, MAPK12, MAPK14, PRKCD, SRC, YES1) significantly inhibited HCT116 cell growth (Fig. 5a, Table S7).

MAPK1 and MAPK3 (ERK2, ERK1, respectively) are kinases that are downstream factors in the EGFR signaling^19^. Thus, a decrease in cell growth by the knockdown of these kinases suggests that one of the resistance mechanisms to cetuximab treatment is the activation of kinases downstream of the EGFR signaling. Activation of ERK1/2-mediated downstream signaling in HCT116 cells was also supported by the results of western blotting, as shown in Fig. 1b.

MAPK13 (p38 delta) is a member of the p38 kinase family^20^. Previously, it has been reported that p38 kinase signaling is important for oncogenesis of colorectal carcinoma and resistance to cetuximab^21^. Therefore, our results obtained from the activity profiling of the kinome are consistent with the published data. This fact suggests that our phosphoproteomic approach could precisely capture phosphorylation signaling, including a known mechanism related to drug resistance in cancer cells.

Intriguingly, knockdown of SRC and PRKCD also decreased cell proliferation in HCT116 cells (Fig. 5a). Our phosphoproteomic approach proposed the SRC-PRKCD cascade as a novel pathway activated in HCT116 cells (Fig. 4a and b). This suggests that the SRC-PRKCD cascade is a novel therapeutic target in Cetuximab-resistant colorectal cancer, and construction of phosphorylation network using phosphoproteomic data will contribute to screening novel kinases as drug targets.

Finally, we used kinase inhibitors to determine if the identified kinases in our study could be drug targets. We treated HCT116 cells with two TKIs targeting SRC (KX2-391) and YES1 (SU6656) and found that both TKIs inhibited not only HCT116 growth but also the growth of other cetuximab-resistant cell lines such as HT29, SW480, and Colo205 (Fig. 5b). Calculation of half-maximal inhibitory concentration (IC_50_) revealed that treatment with an SRC inhibitor, KX2-391, strongly inhibited cell growth of HCT116 (IC_50_: 19 nM), HT29 (IC_50_: 30 nM), SW480 (IC_50_: 36 nM), and Colo205 cells (IC_50_: 29 nM) (Fig. 5b). A YES1 inhibitor, SU6656, also inhibited cell proliferation of HCT116 (IC_50_: 1056 nM), HT29 (IC_50_: 469 nM), SW480 (IC_50_: 1408 nM), and Colo205 cells (IC_50_: 540 nM) (Fig. 5b). Thus, these compounds, especially SRC inhibitors, appear to be promising drugs for overcoming cetuximab resistance in colorectal cancer.

Because the activities of Src family kinases has been reported to be involved in the nuclear translocation of EGFR in Cetuximab-resistant clones^22^, we analyzed this in Cetuximab-resistant cell lines (HCT116 and HT29). We found that EGFR in the nucleus fraction significantly increased in HCT116 (*p* < 0.003) and HT29 (*p* < 0.02) cells after treatment with Cetuximab, although EGFR in the cytosolic fraction was not modulated in both cell lines (Figure S4a, S4b, S4c). Immunocytochemistry showed the nuclear translocation of EGFR in some HCT116 and HT29 cells after treatment with Cetuximab, whereas EGFR clearly localizes in the plasma membrane and cytoplasm of control cells (Figure S5).

## Discussion

In this study, we demonstrated a workflow for identification of activated kinases as druggable targets by using cetuximab-resistant cell lines and deep phospho- and pY proteomic data. We identified activated kinases by their phosphorylation status and by using a KSEA algorithm. We also constructed a network of activated kinases by using activated kinases as network nodes and by connecting them with KSRs. Finally, we validated the roles of the activated kinases in HCT116 proliferation by using siRNAs and chemical inhibitors, which revealed several kinases that have the potential to overcome cetuximab resistance. Our results led us to propose novel therapeutic targets against cetuximab resistance based on the kinome network derived from the results of the phosphoproteomic analysis.

This study demonstrated the importance of phosphoproteomics data for analysis of drug sensitivity. In previous research concerning drug sensitivity in colorectal cancer, genomic analysis was widely applied and revealed many markers, including cetuximab markers. For example, it has been reported that KRAS mutation (e.g., G13D) or BRAF mutation (V600E) changed the sensitivity to cetuximab^23^. However, DLD1, which was placed in the “sensitive group” in this study (Fig. 1b), also contains a resistance-related mutation (G13D) in KRAS. This fact indicated that genomic features could not completely explain the sensitivity to cetuximab. Thus, we aimed to analyze resistance to cetuximab in terms of phosphoproteomic status. The phosphoproteomic approach in this study identified 15 activated kinases that could distinguish between cetuximab-resistant HCT116 cells and two cetuximab-sensitive cell lines. In the COSMIC database, no information on genomic mutation is assigned for 13 of the 15 kinases (Table 1). This fact indicated that the activity profiling of the kinome by using phosphoproteomic approaches could be useful for finding novel companion markers that cannot be determined by genomic approaches. In addition, our results demonstrated that pY proteomic data greatly contributed to characterizing the activity profile of the kinome (Fig. 3c). Further improvement of phosphoproteomic methods would help to obtain a more comprehensive profile of the cellular kinome and greater insights into the relationship between kinase activities and the sensitivity of drugs targeting kinases.

Previously, quantitative proteomics combined with liquid chromatography-tandem mass spectrometry (LC-MS/MS) has also been used in analyses of colorectal cancer resistance to cetuximab^24, 25^. However, information on the protein expression is not always consistent with the enzymatic activities of kinases as potential drug targets. For the purpose of activity profiling of the kinome, reversed-phase protein array (RPPA) has been applied to reveal the activation status of phosphorylation signaling pathways in relation to drug sensitivity^26^. Although RPPA can be used to measure established signaling pathways, it is difficult to obtain comprehensive phosphorylation status, including for unknown pathways, because of the limitations of antibodies. Therefore, in this study, we aimed to measure phosphoproteomic profile with pSTY and pY proteomics and identify unknown targets for overcoming intrinsic resistance to Cetuximab. Indeed, we demonstrated that active kinase candidates are discovered from the deep phosphop- and pY proteomic data (Table 1) and that the Src family kinases, SRC, and YES1 are potential druggable targets in colorectal cancer resistant to Cetuximab as shown in Fig. 5b. These findings indicated that phosphoproteomic data could show direct information on enzymatic activities of kinome and provide novel druggable targets that have not been previously identified from aspects of genomic status or protein expression profiling.

Finally, to interpret regulatory relationship of activated kinases and discuss a mechanism of Cetuximab resistance, we constructed a network of activated kinases using experimentally validated KSRs in PhosphositePlus. We found constitutive activation of the signaling cascade from SRC to PRKCD in HCT116 cells (Fig. 4b). In the curated pathway database KEGG^27^ the regulatory relationship directly from EGFR to SRC is already registered, suggesting that SRC-PRKCD cascade, the downstream pathway of EGFR, may be involved in intrinsic resistance to Cetuximab in HCT116 cells. In addition to SRC, other members of Src family kinases, such as YES1, LYN, HCK, and LCK, were also discovered as potential active kinases in HCT116 cells. Previously, the relationship between EGFR and Src family kinases has been shown in non-small cell lung cancer and colon cancer that are resistant to Cetuximab^22, 28^.

Because activities of Src family kinases are involved in nuclear translocation of EGFR in Cetuximab-resistant clones^22^, we also checked nuclear translocation of EGFR in Cetuximab-resistant cell lines (HCT116 and HT29). Using western blot (Figure S4a, S4b, S4c) and immunocytochemistry (Figure S5), we found that EGFR in the nucleus fraction significantly increased in HCT116 and HT29 cells after treatment with Cetuximab. Previous reports have shown that several kinds of stimuli, such as oxidative stress, irradiation, and treatment with Cetuximab, induce nuclear translocation of EGFR, which is mediated by the activation of Src family kinases^8, 29, 30^. This agreement suggests that the nuclear translocation of EGFR by the activation of Src family kinases might be a general mechanism of endurance to various stresses, and might be beneficial for the expansion of cancer cells that can inherently overcome the stresses involving molecular targeted therapies. Although further study will be needed to clarify molecular mechanism of the intrinsic resistance to Cetuximab and association with Src family kinases, these results suggest that the Src family kinases are novel therapeutic targets in case of Cetuximab-resistant colorectal cancer and construction of phosphorylation network using phosphoproteomic data will contribute to screening novel kinases as drug targets.

Although we demonstrated that knockdown of MAPK13 decreased cell growth in HCT116 cells, we could not construct a regulatory network for MAPK13 when using curated KSRs in a public database because the depth of the currently available KSRs is limited. As shown in Figure S2, >80% of phosphosites could not be annotated with experimentally validated KSRs registered in PhosphositePlus. This limitation causes difficulties in the interpretation of the whole phosphoproteomic data in terms of signaling networks. Therefore, accumulation of more comprehensive KSRs is needed to understand the regulatory functions of whole phosphorylation signaling in cancer. Additionally, statistical methods for network analysis of phosphoproteomic data^31^ or combination of other omics data such as transcriptome^32^ should lead to a better understanding of the cellular phosphorylation signaling.

In summary, our deep phosphoproteomic analysis was found to be very useful for measuring modulation of comprehensive phosphorylation signaling, especially pY signaling. We also demonstrated that a structure of the phosphorylation network constructed from the activity profiling of the kinome was very useful for identifying novel targets for anti-cancer drugs and appears to have good potential for use in future research.

## Material and Methods

### Reagents and antibodies

Lipofectamine RNAiMax, penicillin–streptomycin, a tandem mass tag (TMT) 10-plex isobaric label reagent set, magnetic Dynabeads Protein G for immunoprecipitation, and FBS were obtained from Thermo Fisher Scientific (Waltham, MA, USA). Chemi-Lumi Super and DMEM were purchased from Nacalai Tesque (Kyoto, Japan). KX-391 and SU6656 were purchased from Selleck (Houston, TX, USA). PhosSTOP phosphatase inhibitor cocktails, Trypsin, and cOmplete protease inhibitor cocktails were obtained from Roche (Basel, Switzerland). Tris-buffered saline (TBS) and phosphate-buffered saline (PBS) tablets were obtained from Takara (Shiga, Japan). A detergent compatible (DC) protein assay kit was purchased from Bio-Rad (Hercules, CA, USA). XV Pantera gel (5–20%) for sodium dodecyl sulfate polyacrylamide gel electrophoresis (SDS-PAGE) was purchased from DRC (Tokyo, Japan). RNA-direct SYBR Green RealTime PCR Master Mix was obtained from TOYOBO (Osaka, Japan). The Prism 6 software package was purchased from GraphPad Software (La Jolla, CA, USA). Cell Counting Kit-8 was obtained from Dojindo (Kumamoto, Japan). Cetuximab was a kind gift from Merck KGaA (Darmstadt, Germany). Nuclear/Cytosolic fractionation kit was purchased from Cell Biolabs, Inc. (San Diego, CA, USA).

pMEK1/2 S217/221 (41G9), pERK1/2 T202/Y204 (D13.14.4E), and phospho-tyrosine (P-Tyr-1000) MultiMab antibodies were obtained from Cell Signaling Technology (Danvers, MA, USA). MEK1/2 (9G3), ERK1/2 (MK1) and Lamin A/C antibodies were purchased from SantaCruz (Dallas, TX, USA). GAPDH (6C5) antibody was obtained from Abcam (Cambridge, UK). SRC (327) antibody was obtained from Merck KGaA. pSRC Y418 antibody was purchased from Thermo Fisher Scientific.

### Cell culture and sample collection

DLD1, LIM1215, HT29, HCT116, Colo205, and SW480 cells were cultured at 37 °C under 5% CO_2_. The colorectal cell lines were maintained in DMEM supplemented with 10% FBS and penicillin–streptomycin. The culture media was changed from DMEM with 10% FBS to DMEM with 1% FBS one day before the start of each experiment. The colorectal cells were harvested after washing with ice-cold PBS buffer containing PhosSTOP and cOmplete. Pellets of the collected cells were quickly frozen in liquid nitrogen and stored at −80 °C prior to use. Three biological replicates of each cell lines before or after treatment of Cetuximab were performed for the subsequent phospho- and pY proteomic analysis.

### Preparation of samples for global phosphoproteomics and phosphotyrosine proteomics

Lysis of the cell pellets was performed in phase-transfer surfactant buffer supplemented with cOmplete protease inhibitor cocktail and PhosSTOP, as described previously^14^. The protein concentration was measured with a DC protein assay (Bio-Rad) according to the manufacturer’s protocol. The experimental procedure of pSTY and pY proteomics is summarized in Fig. 1a. A 2.0-mg protein lysate was reduced, alkylated, and subsequently trypsinized, as described previously^33^. The surfactant was removed as described previously^34^. Primary enrichment of phosphopeptides from the 2.0 mg of protein lysate was performed by using Fe^3+^ IMAC resin, as described previously^35^. Phosphopeptides were labeled with the TMT 10-plex reagent according to the manufacturer’s protocol. A correspondence table of each TMT label and each sample is summarized in Figure S3. A total of 20 mg of a labeled phosphopeptide mixture was prepared and then lyophilized. We used 4.0 of the 20 mg of mixture for the fractionation for global phosphoproteomics and used the rest (16 mg) for pY-immunoprecipitation (pY-IP) experiments, respectively. Phosphopeptides for global phosphoproteomics were fractionated into seven fractions by following a previously published protocol^36^. In the experiment of pY-IP, pY peptides were enriched according to the protocol in a previous study^14^.

### LC-MS/MS analysis

LC-MS/MS analysis was performed by using a Q Exactive Plus mass spectrometer (Thermo Scientific) equipped with an UltiMate 3000 Nano LC system (Thermo Scientific) and an HTC-PAL (CTC Analytics, Zwingen, Switzerland). Buffer A (0.1% formic acid, 2% acetonitrile) and buffer B (0.1% formic acid, 90% acetonitrile) were used in the LC mobile phase.

The Q Exactive Plus instrument was operated in data-dependent mode under the following conditions: Heated capillary temperature, 250 °C; spray voltage, 2 kV. More details of the LC-MS/MS analysis are described in the Supplementary methods.

### Data processing for peptide identification

Raw data were processed by using MaxQuant 1.5.1.2 searching against the Uniprot curated human proteome (release 2011_11) combined with 262 common contaminants^37^. More details of MaxQuant parameters are described in the Supplementary methods. Peptides with annotation as “Potential Contaminant” were discarded in subsequent analyses. Phosphosites identified as “Leading proteins” were used for subsequent analyses. The cutoff criteria for selection of the phosphosites were the same as those described previously^38^: Andromeda score, 40; Andromeda delta score, 8; localization probability, 0.75. Of the triplicate experiments, the class 1 phosphosites that were detected at least once are presented in Table S1 (pSTY proteomics) and S2 (pY proteomics). MS Raw files and processed MS files (MaxQuant output files) are available at the jPOST database (http://jpostdb.org/).

### Statistical analysis of identified class 1 phosphosites

Statistical analysis of quantitative data from the phosphoproteomic analysis was performed by using Perseus 1.5.0.31 (www.perseus-framework.org)^39^. Phosphosites with “0” intensities were excluded from subsequent analysis as missing values. The data were log_2_ transformed and normalized by using median centering of the values in each sample. Data obtained from different sets of the TMT mixture were normalized by using reference sample data in each set. For purification of phosphorylation sites showing a significant increase in resistant cell lines, we calculated the fold change and *q* value of identified class 1 phosphosites between cetuximab-sensitive cell lines (N = 6, average of DLD1 and LIM1215 cells) or cetuximab-resistant colorectal cell lines (N = 3, HCT116 cells or HT29 cells). First, we assessed the experimental error in these proteomic analyses and set the cutoff criteria in terms of quantitative differences between the sensitive group and each resistant cell line. We calculated the SD of the fold change between two reference samples (126, 127 N) and the average of the SD in triplicate. The average SD values for the pSTY proteomics and pY proteomics were 0.494 and 0.277, respectively. Increased phosphosites were identified as those with phosphosite expression above 2 SD from the mean, which corresponded to the fold changes of 0.989 (pSTY proteomics) and 0.554 (pY proteomics).

Next, we calculated the *p* value by using a two-tailed Welch’s *t*-test, then adjusted them to the *q* value by using a permutation test. The cutoff criterion of the *q* value was 0.05. Phosphosites that satisfied the above two criteria are presented in Table S3 (pSTY proteomics) and S4 (pY proteomics). The Volcano plots shown in Fig. 2 were generated by using the R package “ggplot2.”

### Profiling of kinome activity and construction of the phosphorylation network in cetuximab-resistant cell

We selected activated kinases by using information on kinase-regulatory phosphosites that were registered in the Uniprot database^16^ and the KSEA results^17^. After identifying significantly increased phosphosites, we selected kinases that showed increased numbers of phosphosites with kinase-regulatory function. For the calculation with KSEA, we used KSRs predicted by the NetworKIN^40^.

For construction of the activated phosphorylation network, we connected activated kinases with experimentally curated KSRs derived from the phosphosites database^15^.

### siRNA/chemical compound treatment, cell growth assay, and calculation of IC_50_

We transfected siRNA with Lipofectamine RNAiMax according to the manufacturer’s instructions. Briefly, we seeded HCT116 cells 24 hours before siRNA transfection. The culture medium was changed to fresh medium with 20 nM siRNA. The HCT116 cells were cultured at 37 °C for 72 hours. When using chemical compounds, we seeded HCT116 cells 24 hours before starting the assay, and changed the culture medium to fresh medium with chemical compounds in each dose.

Then, a cell growth assay was performed by using a Cell Counting Kit-8 according to the manufacturer’s protocol. The results of the siRNA screening are summarized in Table S7. Statistical significance of the siRNA screening was calculated by using the paired Student’s *t*-test (two-tailed), which were adjusted to the *q* value by using the Benjamini–Hochberg method in the R package “p. adjust” function. Curve fitting and calculation of the IC_50_ was performed by using Prism 6.

### Sodium dodecyl sulfate polyacrylamide gel electrophoresis staining gels, western blotting

The SDS-PAGE experiments were performed as described previously^14, 41^. Chemiluminescent measurements of blotted proteins were performed by using Chemi-Lumi Super.

### Quantitative reverse-transcription polymerase chain reaction

Efficiencies of the siRNA knockdown were validated by using qRT-PCR. mRNAs were purified by using an RNeasy Plus Mini Kit (Qiagen, Hilden, Germany) according to the manufacturer’s instruction. The qRT-PCR experiments were performed by using an RNA-direct SYBR Green RealTime PCR Master Mix. The qRT-PCR data were analyzed by using a comparative computed tomographic method and are summarized in Table S6. Sequences of primers for qRT-PCR are listed in Table S8.

### Nuclear and cytosolic fractionation

Colorectal cancer cell lines treated or untreated with 5 μg/ml Cetuximab were fractionated using a Nuclear/Cytosolic fractionation kit according to the manufacturer’s protocol. Protein concentrations in nuclear and cytosolic fraction were measured using a DC protein assay. Next, the fractions were subjected to SDS-PAGE. Lamin A/C and GAPDH were used as nuclear and cytosolic markers, respectively.

### Immunostaining of EGFR in culture cells with or without treatment with Cetuximab

Before treatment with 5 μg/ml Cetuximab, HCT116 and HT29 cells were cultured in 8-well chamber slides (Matsunami glass, Osaka, Japan) for 1 day. After washing with PBS twice, the cells were fixed for 20 min with 4% paraformaldehyde (Merck KGaA) and permeabilized for 15 min with 0.05% Triton-X 100 (Merck KGaA). The samples were blocked for 30 min in 5% bovine serum albumin (BSA) (Merck KGaA) in PBS and then incubated overnight at 4 °C with EGFR antibody (100-fold dilution) in PBS containing 10% FBS and 1% BSA. After washing with PBS 4 times, the samples were incubated for 30 min with secondary antibody conjugated with Alexa Fluor 488 (Abcam) (1000-fold dilution) in PBS containing 10% FBS and 1% BSA. After washing with PBS 4 times, the slides were mounted in ProLong Gold antifade reagent with 4,6-diamidino-2-phenylindole (Thermo Fisher Scientific) and analyzed with a LSM710 confocal microscope (Carl Zeiss, Oberkochen, Germany).

### Accession codes

The accession code for the MS data in this study was JPST000109/PXD005839 in jPOST (http://jpostdb.org/).

## Electronic supplementary material

Supplementary Information

Table S1

Table S2

Table S3

Table S4

Table S5

Table S6

Table S7

Table S8
