# Supplementary material for: Deep Phospho- and Phosphotyrosine Proteomics Identified Active Kinases and Phosphorylation Networks in Colorectal Cancer Cell Lines Resistant to Cetuximab
Source: Sci Rep. 2017 Sep 5;7:10463. doi: 10.1038/s41598-017-10478-9 (PMC5585238; doi:10.1038/s41598-017-10478-9)
Supplement: Supplementary file 1 — Supplementary Information [file 41598_2017_10478_MOESM1_ESM.pdf]

"Deep Phospho-and Phosphotyrosine Proteomics Identified Active kinases and Phosphorylation Networks in Colorectal Cancer Cell Lines Resistant to Cetuximab"

Yuichi Abe[1], Maiko Nagano[1], Takahisa Kuga[1, 2], Asa Tada[1], Junko Isoyama[1], Jun Adachi[1]*, Takeshi Tomonaga[1]*

[1] Laboratory of Proteome Research, National Institute of Biomedical Innovation, Health and Nutrition, Ibaraki, Osaka 567-0085, Japan, [2] Department of Biochemistry and Molecular Biology, Kyoto Pharmaceutical University, Yamashina-ku, Kyoto 607-8414, Japan

Table of Contents

Figure S1 Effect of FBS concentration on cell growth of Cetuximab-treated DLD1 cells.
Figure S2 Percentage of class 1 phosphorylation sites annotated with KSR from PhosphositePlus database.
Figure S3 Correspondence table of each TMT label and each sample
Figure S4 EGFR was translocated into the nucleus after treatment of Cetuximab.
Figure S5 Observation of translocated EGFR into nucleus with immunostaining.
Figure S6 Full-length blots of the data used in Figure 1c.
Figure S7 Full-length blots the data used in Figure 3d.
Figure S8 Full-length blots the data used in Figure S4a.
Table S1 Summary of the class 1 phosphorylation sites detected at least one time of the triplicate experiments in pSTY proteomics.
Table S2 Summary of the class 1 phosphorylation sites detected at least one time of the triplicate experiments in pY proteomics.
Table S3 Summary of phosphorylation sites showing significant increases in HCT116 cells and HT29 cells from pSTY proteomic data.
Table S4 Summary of phosphorylation sites showing significant increases in HCT116 cells and HT29 cells from pY proteomic data.
Table S5 Summary of kinase-regulatory phosphorylation sites showing significant increases in HCT116 cells and HT29 cells.
Table S6 Summary of qRT-PCR experiments containing fold changes in the siRNA screening.
Table S7 Summary of cell growth assays containing fold changes and *q* values in the siRNA screening.
Table S8 Summary of primer sequences and product sizes used for qRT-PCR

Figure S1 Effect of FBS concentration on cell growth of Cetuximab-treated DLD1 cells. Relative cell viability of DLD1 cells at 72 hours after treatment of Cetuximab. Ratio of cell viability was calculated by comparison with the control without treatment of Cetuximab. Error bars show SDs. N = 3.

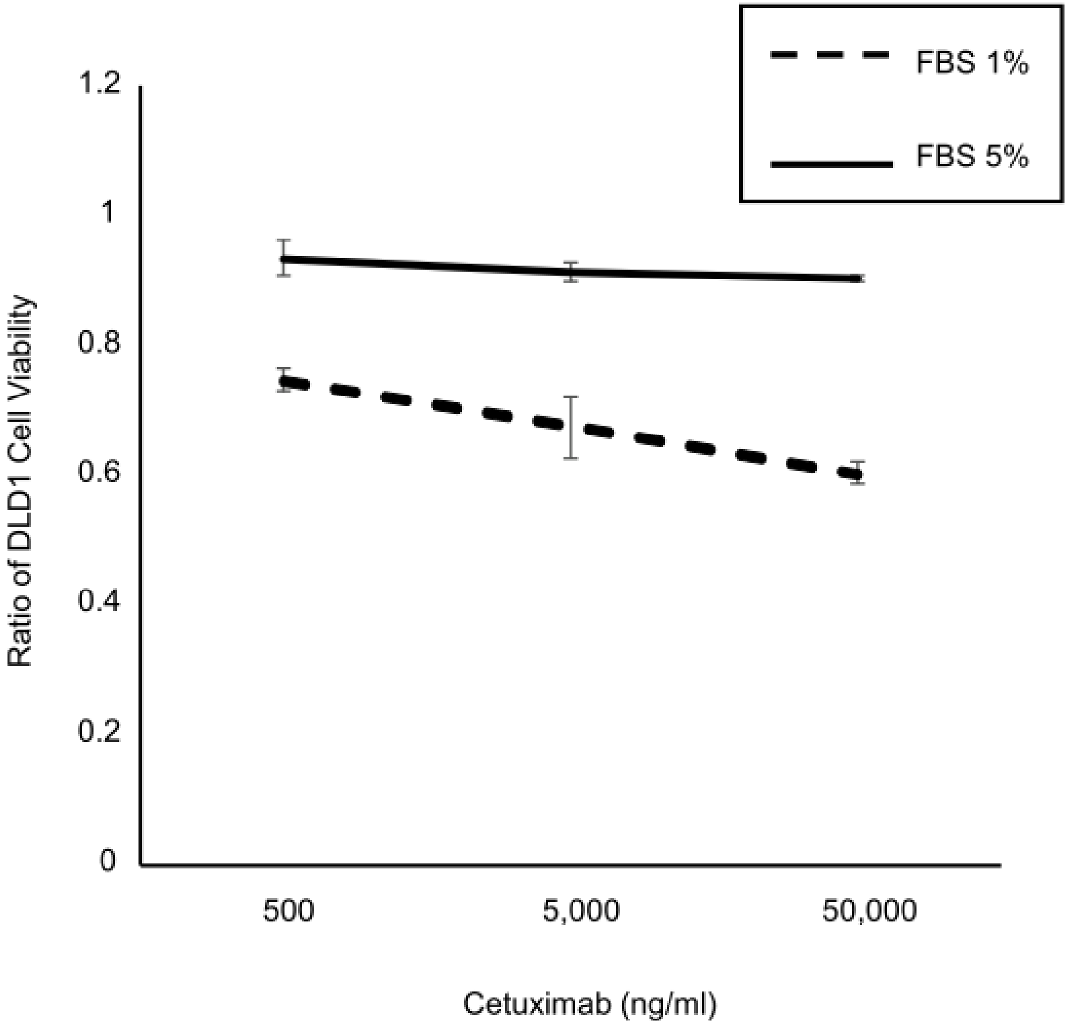

Figure S2 Percentage of class 1 phosphosites annotated with KSR from PhosphositePlus database. Phosphosites detected in all triplicate experiments were annotated with KSR assigned by PhosphositePlus. Gray area shows percentage of phosphosites without KSR annotation. Blue area shows percentage of phosphosites with KSR annotation from PhosphositePlus.

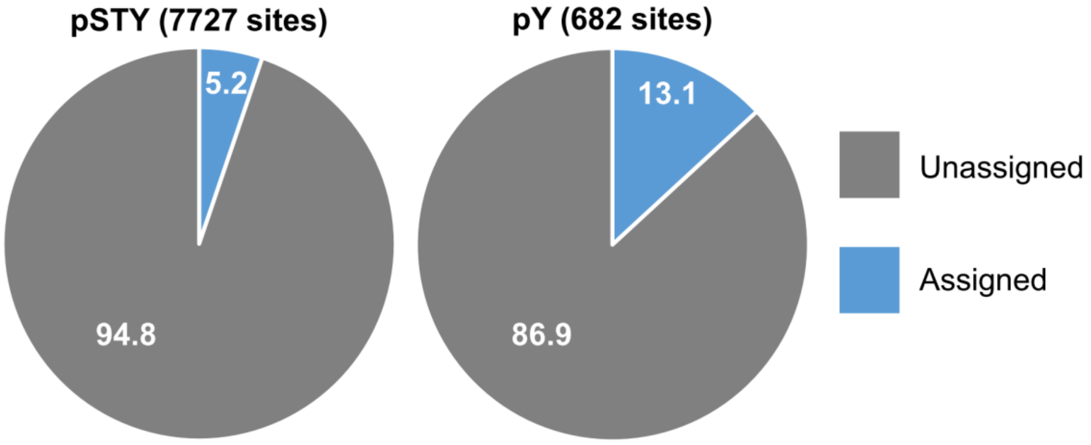

Figure S3 Correspondence table of each TMT label and each sample. "untreated" means samples without treatment of Cetuximab. "treated" means samples with treatment of Cetuximab for 24 hours. "Ref" means a reference sample which is a mixture of other 8 samples (LIM1215 untreated, LIM1215 treated, DLD1 untreated, DLD1 treated, HCT116 untreated, HCT116 treated, HT29 untreated, and HT29 treated) used for deep phospho- phosphotyrosine proteomic analysis.

TMT Set #	126	127N	127C	128N	128C	129N	129C	130N	130C	131
No.1	Ref1	Ref1	LIM1215 untreated	LIM1215 treated	DLD1 untreated	DLD1 treated	HCT116 untreated	HCT116 treated	HT29 untreated	HT29 treated
No.2	Ref2	Ref2	HT29 untreated	HT29 treated	LIM1215 untreated	LIM1215 treated	DLD1 untreated	DLD1 treated	HCT116 untreated	HCT116 treated
No.3	Ref3	Ref3	HCT116 untreated	HCT116 treated	HT29 untreated	HT29 treated	LIM1215 untreated	LIM1215 treated	DLD1 untreated	DLD1 treated

Figure S4 EGFR was translocated into the nucleus after treatment of Cetuximab. (A) Comparison of EGFR translocation into the nuclear fraction in HCT116 and HT29 cells. Lamin A/C is a nuclear marker, and GAPDH is a cytosolic marker. (B) Densitometric analysis of EGFR bands in the nuclear fraction shown in panel A. The densities of protein bands were normalized to Lamin A/C. Error bars show SD. N=3. (C) Densitometric analysis of EGFR bands in the cytosolic fraction shown in panel A. The densities of protein bands were normalized to GAPDH. Error bars show SD. N=3.

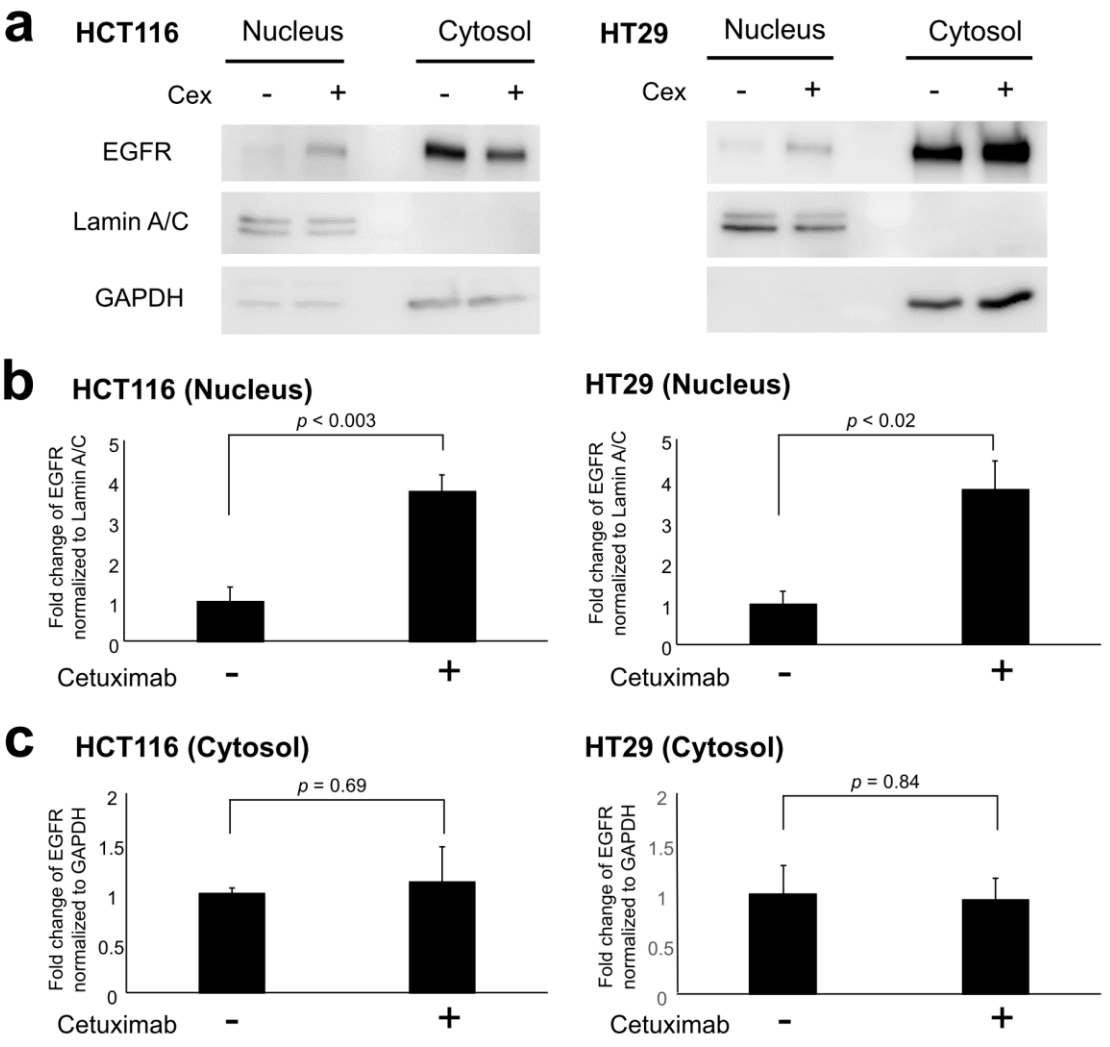

Figure S5 Observation of translocated EGFR into nucleus with immunostaining. Translocation of EGFR (green) into nucleus (blue) was observed in the cells indicated by arrows in HCT116 and HT29 cells. Scale bars, 20 μm.

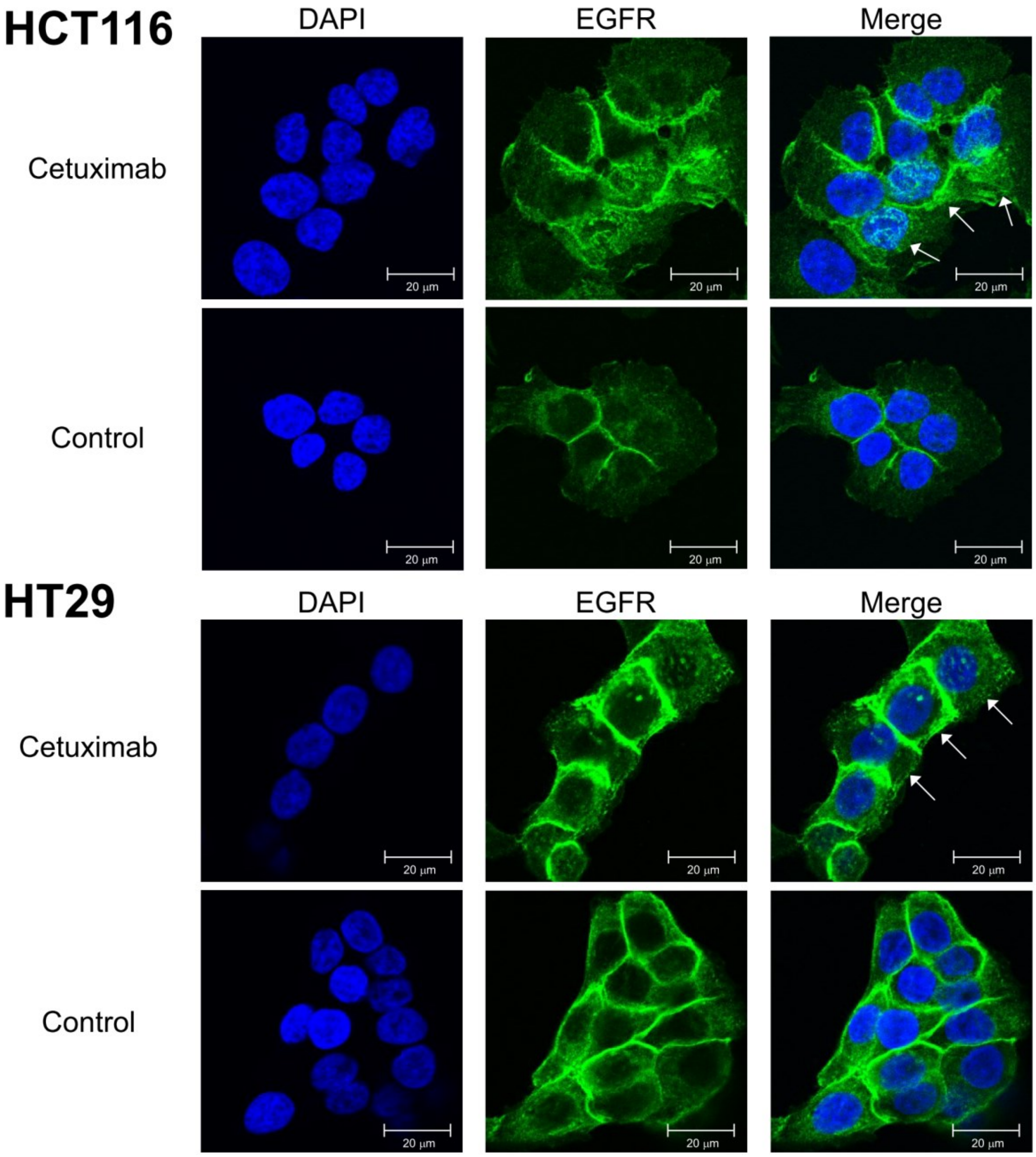

Figure S6 Full-length blots of the data used in Figure 1c.

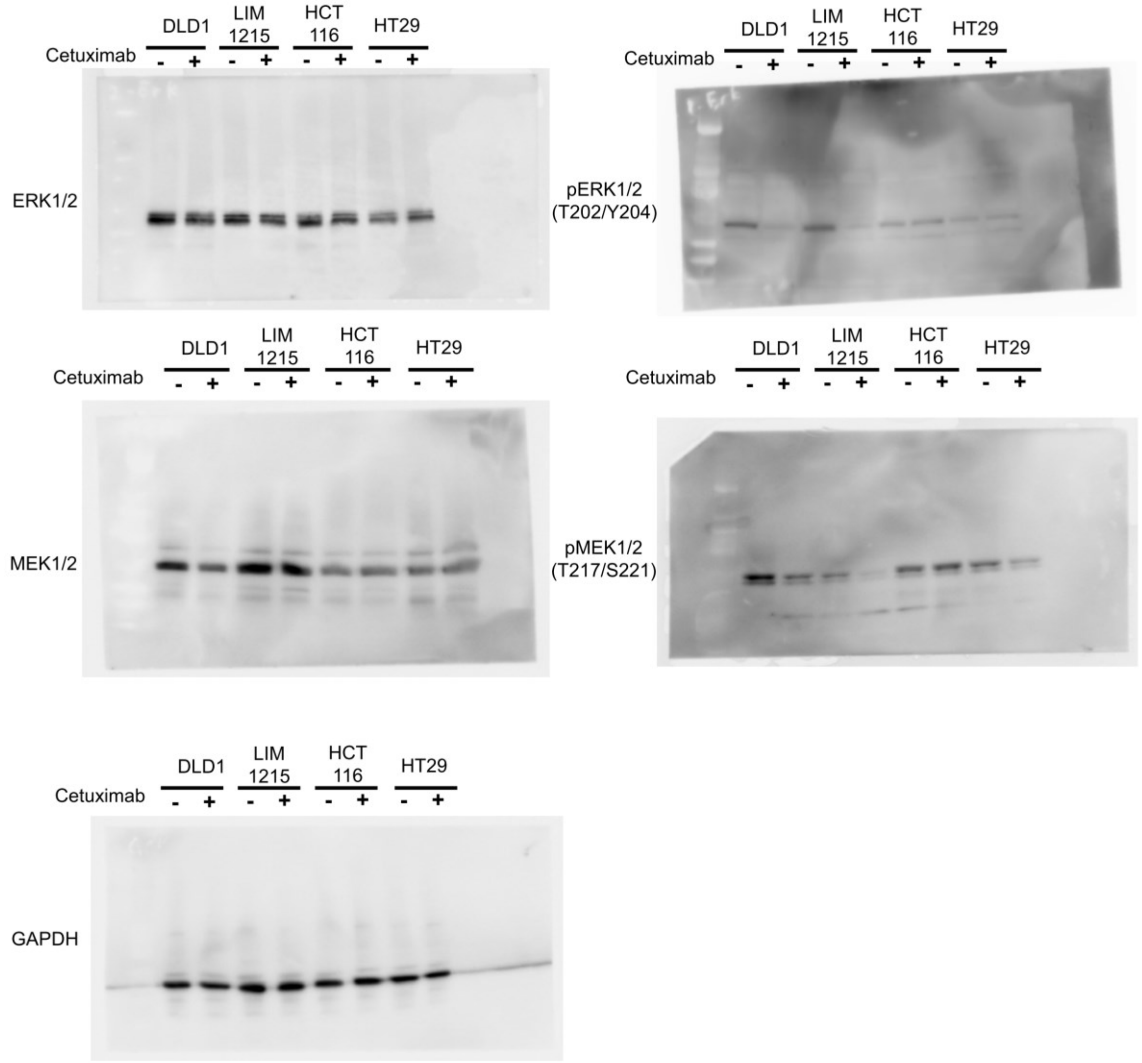

Figure S7 Full-length blots of the data used in Figure 3d.

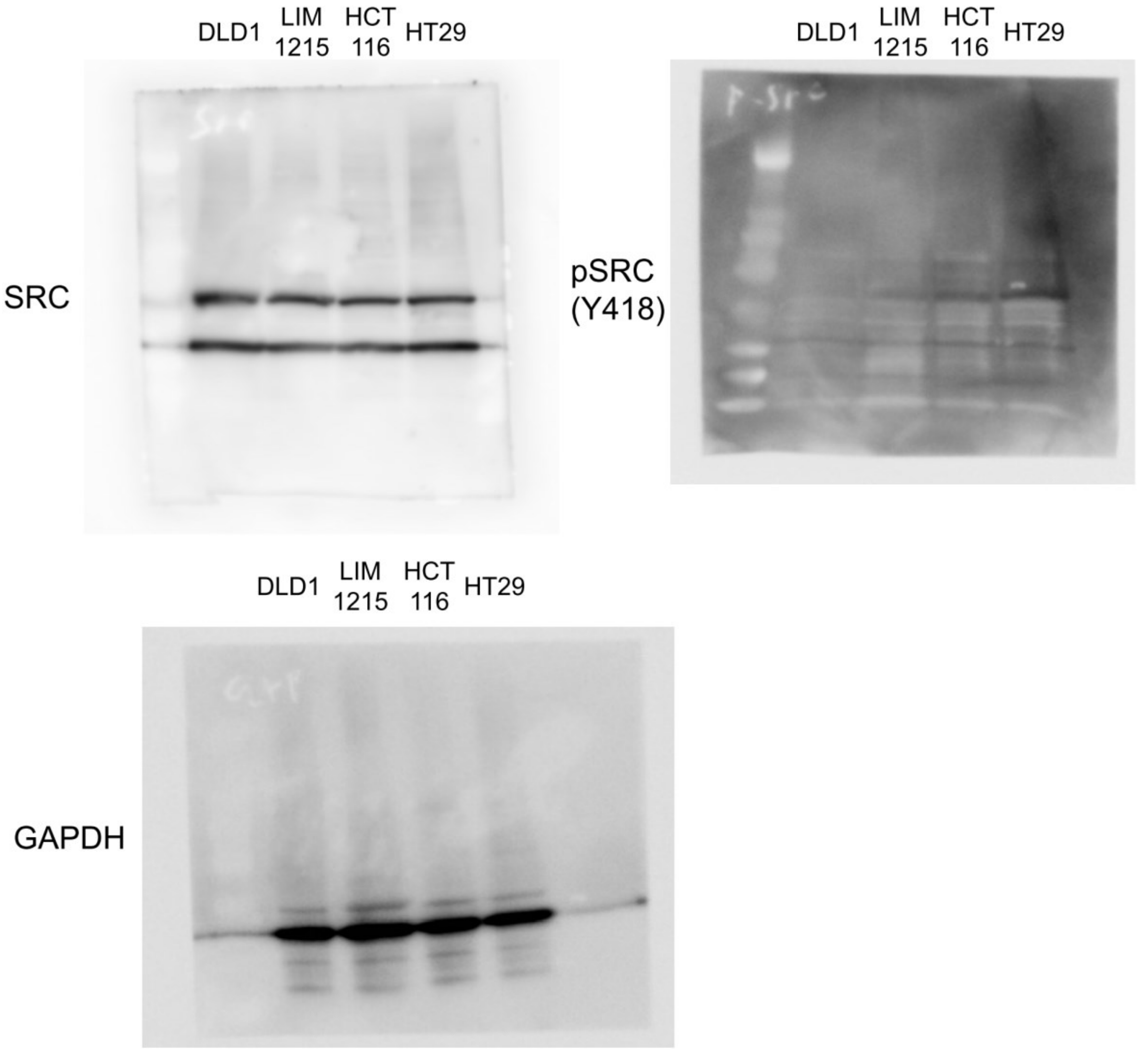

Figure S8 Full-length blots of the data used in Figure S4a. The full-length blot of EGFR in HT29 cells includes the data of “whole cell”, which was not shown in the manuscript.

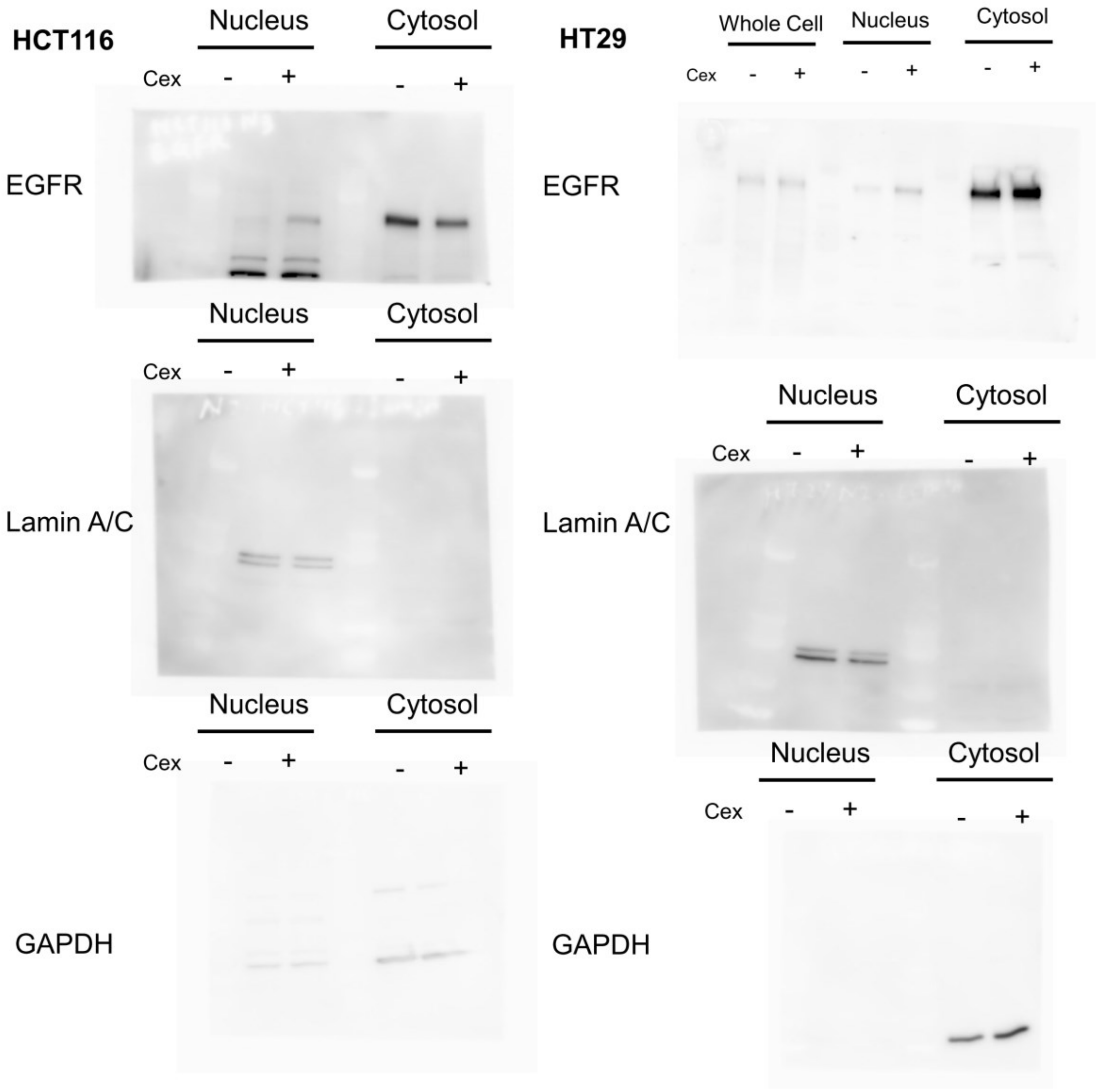

Supplemental Methods

Details of LC-MS/MS analysis

The phosphopeptides in pSTY and pY proteomics were trapped on an Acclaim PepMap RSLC Nano Trap Column (0.1 mm × 20 mm, Thermo Fisher Scientific), then transferred to an analytical column (75 µm × 30 cm, packed with ReproSil-Pur C18-AQ, 1.9 µm resin, Dr. Maisch, Ammerbuch, Germany). The loaded peptides were separated at a flow rate of 280 nL/min using a gradient from 5% to 30% buffer B over 135 min (pSTY proteomics) or 45 min (pY proteomics), respectively.

Survey full scan MS spectra were collected from 350 to 1800 m/z in the Orbitrap with a resolution of 70,000 and an AGC target of 1E6. For the MS/MS experiment, the 12 (pSTY proteomics) or 6 (pY proteomics) most intense multiplied charged precursors ($z \geq 2$) were accumulated to a 1E5 target value and fragmented in the collision cell by higher-energy collisional dissociation (HCD). The precursor isolation width was 2.0 Da (pSTY proteomics) or 3.0 Da (pY proteomics). The HCD normalized collision energy was 25% (pSTY proteomics) or 30% (pY proteomics). The maximum injection times was set to 120 ms (pSTY proteomics) or 240 ms (pY proteomics). The dynamic exclusion was set to 30 s (pSTY proteomics) or 10 s (pY proteomics).

Details of peptide identification with MaxQuant

The following settings were used in the peptide identification with MaxQuant: fixed modification, cysteine carbamidomethyl, TMT tags on lysine residues, and peptide N-termini; variable modification, acetylation on protein N-termini, methionine oxidation, phosphorylation of serine, threonine, and tyrosine; allowed number of missed cleavages, 2; maximum false discovery rate at the protein, peptide, and PTM site levels, 0.01.
